
JOURNAL INFORMATION
==============================
NLM Title Abbreviation: Wirtschaftsdienst
Journal ID: Wirtschaftsdienst
NLM Journal ID: 101086612

Wirtschaftsdienst (Hamburg, Germany : 1949)

ISSN: 0043-6275

ARTICLE INFORMATION
==============================
PMCID: PMC8127501
PMID: 34024954
DOI: 10.1007/s10273-021-2925-2
Article ID: 2925
Article version: 1
Subjects: Ökonomische Trends

Störungen der Geschäftsabläufe durch Corona

Corona disrupts business processes

Grömling Michael groemling@iwkoeln.de
1
Bardt Hubertus bardt@iwkoeln.de
2
Niendorf Paul 2
1 grid.473597.b 0000 0000 9854 4639 Strukturwandel, Verteilung, Lohnfindung, Institut der deutschen Wirtschaft Köln e.V., Konrad-Adenauer-Ufer 21, 50668 Köln, Deutschland
2 grid.473597.b 0000 0000 9854 4639 Institut der deutschen Wirtschaft Köln e.V., Konrad-Adenauer-Ufer 21, 50668 Köln, Deutschland
Electronic publication date: 2021 May 17
Print publication date: 2021
Volume: 101
Issue: 5
First page: 400
Last page: 402
Copyright: © Der/die Autor:in(nen) 2021
License: Open Access: Dieser Artikel wird unter der Creative Commons Namensnennung 4.0 International Lizenz veröffentlicht (https://creativecommons.org/licenses/by/4.0/deed.de).
Open Access wird durch die ZBW — Leibniz-Informationszentrum Wirtschaft gefördert.
License URL: https://creativecommons.org/licenses/by/4.0/

issue-copyright-statement: © The Author(s) 2021

==============================

Die Corona-Pandemie stellt auch im Frühjahr 2021 eine große Herausforderung dar. Aus ökonomischer Sicht wird die Volkswirtschaft auf ihrer Angebots- und Nachfrageseite beeinträchtigt. Auf der Angebotsseite werden Wertschöpfungsketten und Produktionsprozesse gestört, weil ausländische und inländische Zulieferungen – wie bei Halbleitern – stocken und Transportwege eingeschränkt sind. Die Beschäftigten können aufgrund begrenzter Bewegungsfreiheit, geschlossener Bildungseinrichtungen sowie gesundheitlicher Vorsichtsmaßnahmen nicht wie gewohnt arbeiten. Gleichzeitig wird die inländische Nachfrage durch Lockdown-Maßnahmen – wie Teilschließungen des Handels, der Gastronomie und der Kulturwirtschaft – sowie durch eine Investitionszurückhaltung infolge hoher wirtschaftlicher Unsicherheit beschränkt. Das Infektionsgeschehen weltweit belastet auch die Nachfrage nach deutschen Exportgütern, wenngleich sich die deutschen Ausfuhren infolge der schnellen und kräftigen Erholung der Weltwirtschaft gut entwickelt haben.

Im Frühjahr 2021 kämpfen viele Länder mit einer weiteren Infektionswelle. Diese führt zu erneuten Einschränkungen im gesellschaftlichen und wirtschaftlichen Leben. Vor diesem Hintergrund wurden von Ende März bis Mitte April 2021 im Rahmen der Konjunkturumfrage des Instituts der deutschen Wirtschaft (IW) 2.814 Unternehmen in Deutschland nach den aktuellen Risiken für ihre Geschäftsabläufe befragt.

## Störungen der Geschäftsabläufe

Im Rahmen der Zusatzfrage wurde im Frühjahr 2021 – wie im Frühjahr 2020 – nach den Ursachen für Störungen in den Geschäftsabläufen der Firmen gefragt. Die IW-Umfrage liefert damit ein differenziertes Risikoprofil der deutschen Betriebe (vgl. Abbildung 1). Die Antwortmöglichkeiten können in angebotsseitige Störungen und nachfrageseitige Risiken unterteilt werden. Abbildung 2 liefert dazu die Anteile der Firmen in der Abgrenzung Dienstleistungen, Industrie und Baugewerbe, wobei nur die starken Belastungen dargestellt werden.

Angebotsseitig betonen 80 % der Betriebe den Ausfall von Personal – z. B. wegen Krankheit, Betreuung von Angehörigen – als eine Störung ihrer Geschäftsprozesse im Frühjahr 2021. Zwei Drittel aller Firmen sieht dies als ein weniger akutes, 13 % aber als ein starkes Problem. Starke Beeinträchtigungen durch fehlendes Personal werden in den Industrie- und Baufirmen deutlich stärker befürchtet als im Dienstleistungsbereich. Hier können auch betriebsinterne Kommunikationsprobleme, etwa bei Homeoffice oder bestimmten Verwaltungsabläufen, genannt werden. So sprechen 8 % der Firmen von starken und fast 50 % von weniger starken Beeinträchtigungen. Eine starke Betroffenheit sehen Dienstleister und Industrie in etwas höherem Maße als die Baufirmen.

Unsicherheit bezüglich der Lockdown-Maßnahmen, der Öffnungsperspektiven sowie der Standortbedingungen sind für Firmen derzeit eine hohe potenzielle Störung der Geschäftsabläufe. 75 % der Firmen beklagt unklare Lockdown-Maßnahmen – mit fast 40 % weist dieser Punkt den höchsten Wert bei den stark zutreffenden Faktoren auf. Die Betroffenheit ist bei Dienstleistungen im Vergleich zu Industrie und Bau am höchsten. Gut 50 % nennt unsichere Standortbedingungen als eine Geschäftsstörung. Hier sind zusätzliche Regulierungen der Arbeitsbeziehungen, wie Testpflicht am Arbeitsplatz oder das Recht auf Homeoffice, zu nennen. Über alle Branchen hinweg sprechen 18 % von einem hohen Risiko für ihre Betriebsabläufe – in der Industrie sind es sogar 21 %.

Unsicherheit und Volatilitäten in den Wertschöpfungsketten belasten gut 50 % der Firmen. Je nach Argument sprechen rund 15 % bis 20 % von starken Problemen in den Netzwerken. Diese Risiken werden vor allem von der Industrie, aber auch von Baufirmen genannt. Die Verfügbarkeit von inländischen Vorleistungen sowie Einschränkungen bei der Logistik – durch Grenzkontrollen oder fehlende Transportkapazitäten – sehen fast ein Siebtel der Betriebe als starkes und gut

Abbildung 1

**Störung der Geschäftsabläufe im Frühsommer 2021**

Angaben in % der befragten Unternehmen

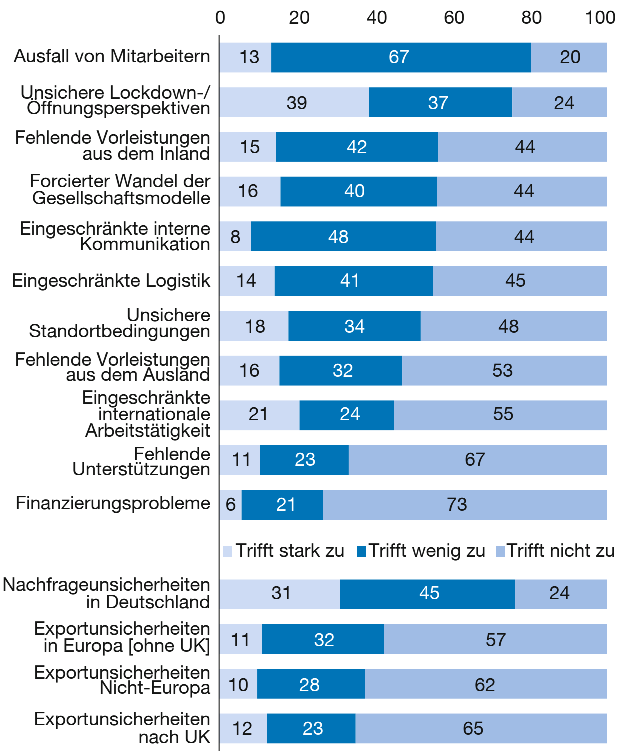

Quelle: IW-Konjunkturumfrage im März/April 2021.

Abbildung 2

**Belastungen nach Branchen im Frühsommer 2021**

Anteil der befragten Unternehmen, die starke Beeinträchtigungen ihrer Geschäftsabläufe durch die folgenden Argumente erwarten in %

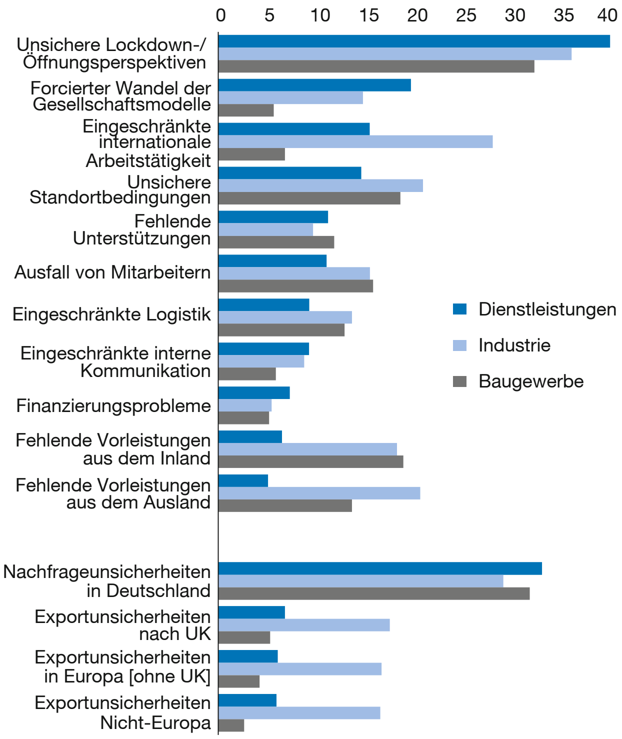

Quelle: IW-Konjunkturumfrage im März/April 2021.

40 % als ein weniger starkes Risiko. Ein Siebtel aller Firmen befürchtet auch starke Produktionsstockungen durch fehlende ausländische Zulieferungen, ein Drittel sieht schwache Beeinträchtigungen. Gut ein Fünftel aller Betriebe moniert die eingeschränkten internationalen Arbeitsmöglichkeiten, da z. B. Monteurdienstleistungen im Ausland, der grenzüberschreitende Mitarbeiteraustausch und Verkaufsgespräche nicht oder kaum möglich sind. Hier ist das Belastungsniveau in der Industrie am höchsten. Es kann davon ausgegangen werden, dass die Pandemie den Strukturwandel – hinsichtlich was und wie produziert wird – sowie die Wertschöpfungskette eines Gutes von Forschung bis Verkauf neu formen und beschleunigen wird (Grömling, 2021). Gut 50 % der Befragten gab an, dass ein forcierter Wandel der Geschäftsmodelle, z. B. durch digitale Konkurrenten, den Betrieb unter Druck setzt. Ein Sechstel sieht dies sogar als starke Herausforderung – bei den Dienstleistern ist es ein Fünftel.

Für fast 75 % der Firmen stellen Finanzierungsprobleme, z. B. durch eingeschränkte Kreditmöglichkeiten, keine Störung der Geschäftsabläufe dar. Im starken Ausmaß betrifft dies nur 6 % aller Betriebe. Fehlende Unterstützungen, wie ausstehende staatliche Unterstützungsleistungen, moniert ein Drittel der Betriebe. Bei 11 % der Firmen stellt dies aktuell ein starkes und branchenübergreifend gleiches Problem dar.

Bei den nachfrageseitigen Belastungen ergibt sich ein gespaltenes Bild: Nur 25 % aller Betriebe konstatiert, dass Absatzunsicherheiten im Inland kein Problem darstellen. Für 45 % stellt dies eine weniger starke Herausforderung dar. Aber für fast ein Drittel sind inländische Nachfragerisiken sogar ein starkes Problem. Das gilt für Dienstleister und Bau etwas stärker als für die Industrie, bei der die Auslandsgeschäfte eine höhere Bedeutung für den Umsatz haben. Exportverunsicherungen stellen für die Industrie eine erheblich höhere Herausforderung dar. Während volatile Auslandsmärkte für deutlich weniger als ein Zehntel der Dienstleister und Baufirmen ein hohes Risiko darstellen, sind es in der Industrie fast ein Fünftel der Betriebe. Unter den Firmen, die eine Orientierung auf globale Märkte angeben, liegt der entsprechende Wert bei ebenfalls fast 20 %. Mit Blick auf alle Firmen sowie die Industrie im Speziellen liefert eine Differenzierung nach europäischem Markt und dem Handel mit nicht-europäischen Ländern keine nennenswert abweichenden Befunde.

## Abgleich mit Frühjahr 2020

Auch im Frühjahr 2020 wurden die Auswirkungen der Pandemie auf die Geschäftsabläufe der Firmen ermittelt (Bardt und Grömling, 2020). Es zeigten sich keine relevanten Unterschiede in der Einschätzung der Industrie- und Dienstleistungsbetriebe. Im Gegensatz zur Finanzmarktkrise 2009 und zur aktuellen Lage wurden nach Ausbruch der Pandemie die Erwartungen der Industrie- und Dienstleistungsfirmen zu Anfang gleichermaßen stark beeinträchtigt. Deutlich schwächer waren die erwarteten Belastungen im Baugewerbe.

Bei den Erwartungen stand im Frühjahr 2020 vor allem der Nachfrageschock im Vordergrund, – wobei sich die negativen Einschätzungen im Zeitverlauf verringerten. Nach den Anfangs hohen Werten belief sich der Anteil der Firmen, die starke Nachfrageeinschränkungen im Inland im Frühjahr/Sommer 2020 erwarteten, relativ konstant auf rund 45 %. Der Anteil der Firmen, die kurzfristig eine starke Beeinträchtigung der Auslandsnachfrage sahen, lag relativ konstant bei gut 25 %. Diese Werte sind deutlich höher als im Frühjahr 2021.

Daneben waren fehlende importierte Vorleistungen die größte Angebotsstörung. Gut 25 % der Firmen sah sich davon stark, weitere 45 % schwach betroffen. Hier ist die aktuelle Lage deutlich entspannter. Der Produktionsausfall durch fehlende inländische Vorleistungen war für weniger als ein Fünftel ein starkes und gut 40 % der Betriebe ein schwaches Problem. Dies liegt nahe an den aktuellen Werten. Durch fehlende Mitarbeiter:innen sahen sich im Frühjahr 2020 rund ein Siebtel stark und fast 50 % der Firmen schwach beeinträchtigt. Das entspricht nahezu der aktuellen Risikoeinschätzung. Knapp ein Fünftel der Firmen sahen akute und starke Finanzierungsschwierigkeiten. Der Anteil der Betriebe, die 2020 mit starken Finanzierungsproblemen bis Ende 2021 rechneten, lag in ähnlicher Größenordnung, was aus der damaligen Sicht der Firmen auf eine nicht nur kurzfristig notwendige Liquiditätssicherung hindeutete. Laut der aktuellen IW-Umfrage hat sich das Finanzierungsrisiko reduziert.

### Reaktionsmöglichkeiten auf Unternehmensebene

Die vielen Ursachen für Störungen des Geschäftsablaufs stellen Firmen vor vielfältige Aufgaben. Dazu gehören Maßnahmen des Arbeitsschutzes und Testangebote zum Schutz vor Ansteckungen ebenso wie Notfallkonzepte, die sowohl lang- wie auch kurzfristig eine klare Orientierung geben (Nohria, 2020; Deloitte, 2020). Aufgrund des zunehmenden Wissens über Ansteckungswege und Schutzmöglichkeiten sind Aktualisierungen notwendig. Andere Restriktionen sind schwieriger zu überwinden. Dienstreisen werden zwar teilweise durch Videokonferenzen ersetzt, die hohe Bedeutung dieser Probleme zeigt jedoch, dass die Ersatzmöglichkeiten an Grenzen stoßen und Einschränkungen für die Geschäftstätigkeit verbleiben. Im Fokus der Diskussion stehen oftmals Probleme in den Wertschöpfungsketten, da Vorprodukte nicht oder nur eingeschränkt lieferbar sind. Firmen können verschiedene Maßnahmen treffen, um ihre Lieferketten resilienter zu gestalten (Biedermann und Kotzab, 2019, 2021).

- Mittels *Monitoringsystemen* in Echtzeit mit automatisiertem Datenaustausch zwischen allen Firmen der Lieferkette können Betriebe Störungen frühestmöglich erkennen und Gegenmaßnahmen einleiten. Dies verstärkt vor allem die Agilität und reaktiven Kapazitäten der jeweiligen Lieferkette. Eine ausreichende und qualitativ hochwertige Datengrundlage würde zudem die Nutzung von KI im Lieferkettenrisikomanagement ermöglichen (Baryannis et al., 2019).

- Firmen sollten in- und ausländische *Kompensationsmöglichkeiten* betrachten. Deutschland ist in einigen Vorleistungsgütergruppen selbst Netto-Exporteur und es gibt zudem auf dem Weltmarkt oftmals mehrere Alternativen für die meisten Vorleistungsgütergruppen (vbw, 2020). Eine Diversifizierung der Lieferketten und eine Analyse strategischer Abhängigkeiten kann die Resilienz von Firmen erhöhen, dabei muss der Tradeoff zwischen Kosten und Risiko abgewogen werden (Kolev und Obst, 2020).

- Die Verbesserung der *Resilienz* der Lieferketten muss Teil der langfristigen strategischen Planung sein. Es gilt vor allem unternehmensübergreifende Organisationsstrukturen zu etablieren, um die Zusammenarbeit mit den Beteiligten effizient zu gestalten. Dazu gehört ein etabliertes Krisenkonzept, das wichtige Zulieferer einschließt und Unsicherheit bei Disruptionen mindert. Resilienz bedeutet nicht nur, dass der Eintritt bestimmter Risiken vermieden werden soll, sondern, dass im Falle eines Schadens eine schnelle Rückkehr zur vollen Produktion möglich ist.

Prof. Dr. Michael Grömling leitet die Forschungsgruppe Konjunktur am Institut der deutschen Wirtschaft (IW) in Köln.

Dr. Hubertus Bardt leitet den Bereich Wissenschaft am IW in Köln und ist Lehrbeauftragter an der Heinrich-Heine-Universität Düsseldorf.

Paul Niendorf ist Working Student beim Institut der deutschen Wirtschaft (IW) in Köln.


Literatur

Bardt H Grömling M Kein schnelles Ende des Corona-Schocks: Ökonomische Einschätzungen deutscher Unternehmen IW-Trends 2020 47 2 21 41
Baryannis G Validi S Dani S Antoniou G Supply Chain Risk Management and Artificial Intelligence: State of the Art and Future Research Directions International Journal of Production Research 2019 57 7 2179 2202 10.1080/00207543.2018.1530476
Biedermann, L. und H. Kotzab (2019), Erfolgsfaktoren zur zukünftigen Gestaltung resilienter Supply Chains — Konzeption eines Bezugsrahmens, in C. Bode, R. Bogaschewsky, M. Eßig, R. Lasch und W. Stölzle (Hrsg.), Supply Management Research. Advanced Studies in Supply Management, 240–254.
Biedermann L Kotzab H Supply Chain Risk Management und Supply Chain Resilienz WiSt — Wirtschaftswissenschaftliches Studium 2021 50 4 4 12 10.15358/0340-1650-2021-4-4
Deloitte (2020), Increasing Organizational resilience, in the face of COVID 19.
Grömling M COVID-19 and the Growth Potential Intereconomics 2021 56 1 45 49 10.1007/s10272-021-0950-4 33518789 PMC7836349
Kolev, G. und T. Obst (2020), Die Abhängigkeit der deutschen Wirtschaft von internationalen Lieferketten, IW-Report, 16.
Nohria, N. (2020), What Organizations Need to Survive a Pandemic, Harvard Business Review.
vbw (2020), Verbesserung der Resilienz der bayerischen Wirtschaft.
